# Bariatric-Metabolic Surgery Utilisation in Patients With and Without Diabetes: Data from the IFSO Global Registry 2015–2018

**DOI:** 10.1007/s11695-021-05280-6

**Published:** 2021-02-27

**Authors:** Richard Welbourn, Marianne Hollyman, Robin Kinsman, John Dixon, Ricardo Cohen, John Morton, Amir Ghaferi, Kelvin Higa, Johan Ottosson, Francois Pattou, Salman Al-Sabah, Merhan Anvari, Jacques Himpens, Ronald Liem, Villy Våge, Peter Walton, Wendy Brown, Lilian Kow

**Affiliations:** 1 Department Upper GI and Bariatric Surgery, Musgrove Park Hospital, Taunton, TA1 5DA UK; 2 Dendrite Clinical Systems Ltd., Henley-on-Thames, Oxfordshire RG9 1AY UK; 3 Iverson Health Innovation Research Institute, Swinburne University, Melbourne, Australia; 4 The Center for Obesity and Diabetes, Oswaldo Cruz German Hospital, São Paulo, Brazil; 5 Division Chief, Bariatric and Minimally Invasive Surgery, Yale School of Medicine, New Haven, CT USA; 6 Department of Surgery, University of Michigan, Ann Arbor, MI USA; 7 UCSF, Fresno, CA USA; 8 Department of Surgery, Faculty of Medicine and Health, Örebro University, Orebro, Sweden; 9 Faculty of Medicine, University of Lille, Lille, France; 10 Al-Amiri Hospital Kuwait, Royale Hyatt Hospital, Kuwait City, Kuwait; 11 Department of Surgery, McMaster University, Hamilton, Ontario Canada; 12 CHIREC Delta Hospital, Brussels, Belgium; 13 Department of Surgery, Groene Hart Hospital, Gouda, Netherlands; 14 Scandinavian Obesity Surgery Registry, Bergen, Norway; 15 Centre of Obesity Research and Education, Monash University, Melbourne, Australia; 16 College of Medicine and Public Health, Flinders University, Adelaide, Australia

**Keywords:** Obesity surgery, Bariatric surgery, Demographic classification, Comorbidity, Sex characteristics, Type 2 diabetes, IFSO Global Registry, Operation choice for diabetes, Metabolic surgery

## Abstract

**Background:**

Comparative international practice of patients undergoing bariatric-metabolic surgery for type 2 diabetes mellitus (T2DM) is unknown. We aimed to ascertain baseline age, sex, body mass index (BMI) and types of operations performed for patients with T2DM submitted to the IFSO Global Registry.

**Materials and Methods:**

Cross-sectional analysis of patients having primary surgery in 2015–2018 for countries with ≥90% T2DM data completion and ≥ 1000 submitted records.

**Results:**

Fifteen countries including 11 national registries met the inclusion criteria. The rate of T2DM was 24.2% (99,537 of 411,581 patients, country range 12.0–55.1%) and 77.1% of all patients were women. In every country, patients with T2DM were older than those without T2DM (overall mean age 49.2 [SD 11.4] years vs 41.8 [11.9] years, all *p* < 0.001). Men were more likely to have T2DM than women, odds ratio (OR) 1.68 (95% CI 1.65–1.71), *p* < 0.001. Men showed higher rates of T2DM for BMI <35 kg/m^2^ compared to BMI ≥35.0 kg/m^2^, OR 2.76 (2.52–3.03), *p* < 0.001. This was not seen in women, OR 0.78 (0.73–0.83), *p* < 0.001. Sleeve gastrectomy was the commonest operation overall, but less frequent for patients with T2DM, patients with T2DM 54.9% vs without T2DM 65.8%, OR 0.63 (0.63–0.64), *p* < 0.001. Twelve out of 15 countries had higher proportions of gastric bypass compared to non-bypass operations for T2DM, OR 1.70 (1.67–1.72), *p* < 0.001.

**Conclusion:**

Patients with T2DM had different characteristics to those without T2DM. Older men were more likely to have T2DM, with higher rates of BMI <35 kg/m^2^ and increased likelihood of food rerouting operations.

**Supplementary Information:**

The online version contains supplementary material available at 10.1007/s11695-021-05280-6.

## Introduction

Over the last decade, randomised controlled trials (RCTs) [1–5], systematic reviews, meta-analyses [6, 7] and international guidelines [8–11] have indicated the benefits of bariatric-metabolic surgery for patients with type 2 diabetes mellitus (T2DM) and severe obesity compared to medical therapy alone. ‘Bariatric’ surgery that produces weight loss overlaps with ‘metabolic’ or ‘diabetes’ surgery where the aim is to improve conditions such as T2DM [12]. The term metabolic surgery has been defined as ‘the operative manipulation of a normal organ or organ system to achieve a biological result for a potential health gain’, and has come to embrace any intervention of the gastrointestinal tract that improves T2DM, regardless of baseline body mass index (BMI) [13], while bariatric surgery, from the Greek ‘baros’ weight or pressure, and ‘-iatric’, the medicine or surgery thereof, has weight loss and its associated benefit as the primary endpoint. ‘Bariatric-metabolic surgery’ is now commonly used to denote the entirety of this area of surgery.

Bariatric-metabolic surgery also improves a range of other obesity-related diseases, provides survival benefit and is cost effective [14, 15]. Despite many international bariatric surgical societies also adding the word ‘metabolic’ to their names to emphasise these positive effects of the procedures, the penetrance of bariatric-metabolic surgery continues to be very low compared to the large number of people who might benefit [16].

There are few comparative data on which patients worldwide are receiving bariatric-metabolic surgery [17, 18]. Mapping current international practices could provide a baseline for strategies to increase its availability and uptake. The International Federation of Surgery for Obesity (IFSO) has undertaken several surveys, mostly relying on estimates, over the last 20 years [19–23]. These reports were able to describe only operation type and procedure numbers, without details on demography or obesity-related disease. A description of which patients with T2DM are receiving this surgery on an international basis and whether having T2DM influences the procedure undertaken is currently lacking.

In 2014, IFSO established a Global Registry project partly to fill these knowledge gaps [24]. The 5^th^, 2019 report, contained descriptive information for 833,687 anonymised individual patient records from 61 countries including 17 national registries, 25 multi-centre submissions and 19 single centres accumulated since its inception [25]. So far 2 reports have described broad characteristics for the submitted data [17, 18]. The Global Registry can potentially provide a detailed description of uptake of bariatric-metabolic surgery for T2DM. We hypothesised that countries with higher prevalence of disease would have a greater proportion of operated patients with T2DM.

This study aimed to describe the differences in demographic data and type of bariatric and metabolic surgery performed in patients with and without T2DM according to the IFSO Global Registry 2015–2018. A secondary aim was to estimate the relative rates of surgery performed for patients with T2DM by comparison to T2DM prevalence in each country’s general adult population.

## Materials and Methods

### Study Design and Participants

A cross-sectional study was performed of the baseline data for patients with or without T2DM having primary bariatric-metabolic surgery from the IFSO Global Registry 2019 data cut. STROBE guidelines were followed. A certificate of exemption from NHS Research Ethics Committee (REC) approval for the study was obtained from the UK Human Research Authority decision tools available on the website http://www.hra-decisiontools.org.uk/ethics/. Countries were chosen that had ≥90% complete baseline T2DM data and ≥ 1000 individual anonymised patient records for the calendar years 2015–2018. As this was an observational study it was not powered to detect a specified difference between analysed groups.

Data were uploaded either individually by each submitting centre or by an upload from the national registry as previously described [17, 18]. The contributors were reassured that no statistical comparison would be attempted between different countries for outcomes that would differentiate quality such as complications or mortality.

Data for individual country prevalence of T2DM were accessed from the Non-Communicable Diseases Risk Factor Collaboration (NCD-RisC) for adults aged 18 years or over using 2014, the latest available year data [26]. These provided a basis for estimating whether the proportion of patients who had T2DM at the time of operation varied according to country disease prevalence. Data for T2DM prevalence for the same age and BMI range as those patients presenting for bariatric-metabolic surgery in each country were not available.

### Procedures

The procedures in the data set (version 4.1) were as described previously and comprised gastric band/gastric bypass/sleeve gastrectomy/duodenal switch/duodenal switch with sleeve/biliopancreatic diversion with sleeve/biliopancreatic diversion/other, and type of gastric bypass: Roux-en-Y (RYGB) or one anastomosis (OAGB) [17, 18].

### Outcomes

Other variables collected were age or date of birth, sex, height, weight, T2DM defined as being on medication yes/no. Only valid records, defined as those including height, weight and calculated BMI, were included for analysis. Data were grouped according to T2DM on medication, age, sex and BMI. The BMI groups were stratified according to obesity severity <35.0 (class I), 35.0–39.9 (class II), 40.0–49.9 (class III), and > 49.9 kg/m^2^. Types of operation were assessed to investigate practice undertaken for T2DM according to BMI groupings.

### Statistical Analysis

After skewness and kurtosis testing, continuous data were described by means (standard deviation), means (95% confidence intervals) and compared by independent sample *t* test. Categorical data were compared by *χ*^2^ with Bonferroni correction for multiple comparisons and odds ratios (ORs) with 95% CIs. Where multiple comparisons were made, *p* < 0.003 was taken to indicate statistical significance. A sensitivity analysis was performed to adjust for the potential dominant effect of the USA on outcomes as 72% of all data were from this country.

## Results

Forty-six of 61 countries that contributed data to the Global Registry 2015–2018 had completion rates of ≥90% for baseline T2DM status, and 26 countries submitted ≥1000 records. Fifteen countries met the inclusion criteria and had data available for further analysis, including 11 of the 14 contributing national registries, comprising 69.5% (413,048) of the 594,235 operation records for the date range (2015–2018) of the 5th IFSO report. The mean baseline T2DM data completion rate was 99.6% (country range 93.7–100%). The number on medication for T2DM was 99,537 of 411,581 (24.2%, country range 12.0–55.1%) and 77.1% of the overall population was female. The total numbers of operations, numbers per country with T2DM, rates of female patients and BMI are shown (Tables 1 and 2). No sex data were available for Chile. OAGB was not separately identified in the USA during the study period.

**Table 1:** Number of contributing hospitals and patients, percentage female, data completion rate and patients on medication for T2DM^a^

Country	Hospitals contributing	Number of patients	Percentage female	Data completion rate for T2DM	Patients on medication for T2DM			
					Overall	Male	Female	*p* value *
**Austria**	14	2211	71.1% (1553/2184)	100.0%	55.1% (1219/2211)	60.5% (382/631)	53.6% (832/1553)	0.003
Bahrain	2	1622	64.9% (1052/1622)	100.0%	21.5% (349/1622)	20.7% (118/570)	22.0% (231/1052)	0.56
**Brazil**	28	1205	68.4% (800/1170)	97.1%	14.1% (165/1170)	19.2% (71/370)	11.8% (94/800)	<0.001
Chile	3	1116	N/A	96.0%	20.0% (214/1071)	N/A	N/A	N/A
**Egypt**	45	3450	66.2% (2139/3233)	93.7%	15.0% (486/3233)	16.5% (181/1094)	14.3% (305/2139)	0.085
**France**	80	8239	77.1% (6153/7979)	96.8%	12.3% (979/7979)	20.5% (375/1826)	9.8% (604/6153)	<0.001
**India**	51	11,806	57.7% (6675/11,569)	98.0%	28.1% (3256/11,569)	32.7% (1599/4894)	24.8% (1657/6675)	<0.001
**Israel**	34	27,292	68.0% (18,541/27,262)	99.9%	16.0% (4371/27,262)	22.6% (1967/8721)	13.0% (2404/18,541)	<0.001
**Kuwait**	6	2651	72.9% (1863/2555)	96.7%	12.8% (329/2563)	15.6% (108/692)	11.8% (219/1863)	0.010
Qatar	2	4734	66.3% (3139/4732)	100.0%	16.8% (793/4734)	16.6% (265/1593)	16.8% (527/3139)	0.90
**Russia**	25	4254	74.9% (3165/4227)	99.4%	15.9% (672/4227)	24.5% (260/1062)	13.0% (412/3165)	<0.001
**Sweden**	42	20,717	77.6% (16,069/20,705)	99.9%	12.0% (2483/20,705)	21.2% (983/4636)	9.3% (1500/16,069)	<0.001
United Arab Emirates	6	1092	62.3% (679/1090)	100.0%	29.0% (317/1092)	26.8% (110/411)	30.3% (206/679)	0.21
**United Kingdom**	168	25,897	78.8% (19,992/25,381)	98.0%	23.0% (5825/25,381)	36.1% (1946/5389)	19.4% (3879/19,992)	<0.001
**United States of America**	Not known	296,762	79.1% (234,783/296,762)	100.0%	26.3% (78,079/296,762)	35.0% (21,664/61,979)	24.0% (56,415/234,783)	<0.001
All		413,048	77.1% (316,603/410,471)	99.6%	24.2% (99,537/411,581)	32.0% (30,029/93,868)	21.9% (69,285/316,603)	<0.001

^a^Bold indicates national registry. T2DM type 2 diabetes mellitus, N/A not available. Note denominators vary due to data completion rate. *p value denotes proportion of females vs males with/without medication for T2DM, chi^2^ test

**Table 2:** Mean age and BMI for patients with or without medication for T2DM^a^

	Age (mean (SD), y)			Initial BMI (mean (SD), kg/m^2^)		
Country	Patients not on medication for T2DM	Patients on medication for T2DM	*p* value	Patients not on medication for T2DM	Patients on medication for T2DM	*p* value
**Austria**	37.1 (12.1)	40.8 (12.5)	<0.001	44.6 (7.1)	45.1 (6.9)	0.15
Bahrain	33.0 (9.7)	42.3 (11.0)	<0.001	46.2 (7.8)	45.0 (9.9)	0.017
**Brazil**	36.8 (10.0)	45.5 (11.2)	<0.001	41.5 (6.8)	40.8 (6.4)	0.22
Chile	38.5 (9.4)	46.5 (8.7)	<0.001	37.2 (3.9)	36.5 (4.1)	0.029
**Egypt**	34.6 (10.4)	44.2 (9.5)	<0.001	46.0 (8.2)	48.4 (9.2)	< 0.001
**France**	40.3 (11.8)	50.8 (10.5)	<0.001	41.9 (6.0)	42.9 (6.4)	< 0.001
**India**	39.6 (12.3)	48.9 (10.7)	<0.001	44.0 (7.8)	43.0 (8.0)	< 0.001
**Israel**	38.5 (12.1)	50.7 (10.9)	<0.001	42.2 (4.8)	41.3 (5.8)	< 0.001
**Kuwait**	31.3 (10.4)	42.8 (11.3)	<0.001	44.0 (7.5)	44.3 (8.4)	0.44
Qatar	31.5 (10.9)	34.6 (12.1)	<0.001	42.9 (6.0)	42.1 (6.0)	0.001
**Russia**	39.9 (10.4)	49.3 (9.5)	<0.001	45.3 (9.3)	46.9 (9.6)	< 0.001
**Sweden**	39.8 (11.2)	48.2 (10.4)	<0.001	41.0 (5.8)	41.2 (5.8)	0.089
United Arab Emirates	33.2 (9.8)	39.5 (10.5)	<0.001	43.9 (5.9)	42.3 (6.6)	0.000
**United Kingdom**	43.5 (11.3)	50.4 (10.0)	<0.001	46.8 (8.0)	46.6 (7.8)	0.13
**United States of America**	42.9 (11.7)	49.5 (11.4)	<0.001	47.4 (8.3)	47.8 (8.8)	< 0.001
All	41.8 (11.9)	49.2 (11.4)	<0.001	46.2 (8.1)	46.9 (8.7)	< 0.001

^a^Bold indicates national registry. T2DM type 2 diabetes mellitus. *p* value independent sample *t* test

### Demographic Characteristics of Those with and Without T2DM

In every country, patients with T2DM were older than those without T2DM (overall mean age 49.2 (11.4) years vs 41.8 (11.9) years, all *p* < 0.001) (Table 2). Men were older than women irrespective of T2DM status. The mean ages of men and women with T2DM were 50.9 (11.1) years and 48.5 (11.5) years respectively, *p* < 0.001, and for those without T2DM 42.3 (12.1) years and 41.8 (11.8) years respectively, *p* < 0.001.

The overall BMI was slightly higher for those with T2DM (mean 46.9 (8.7) kg/m^2^ vs 46.2 (8.1) kg/m^2^, *p* < 0.001). However, the BMI was higher in only 4 countries including the USA, the biggest contributor to the dataset, lower in 4 countries, and similar in 7 countries (Table 2). The majority of all patients with T2DM were in the BMI range 40.0–49.9 kg/m^2^, despite the older age of those with T2DM.

**Fig. 1:**
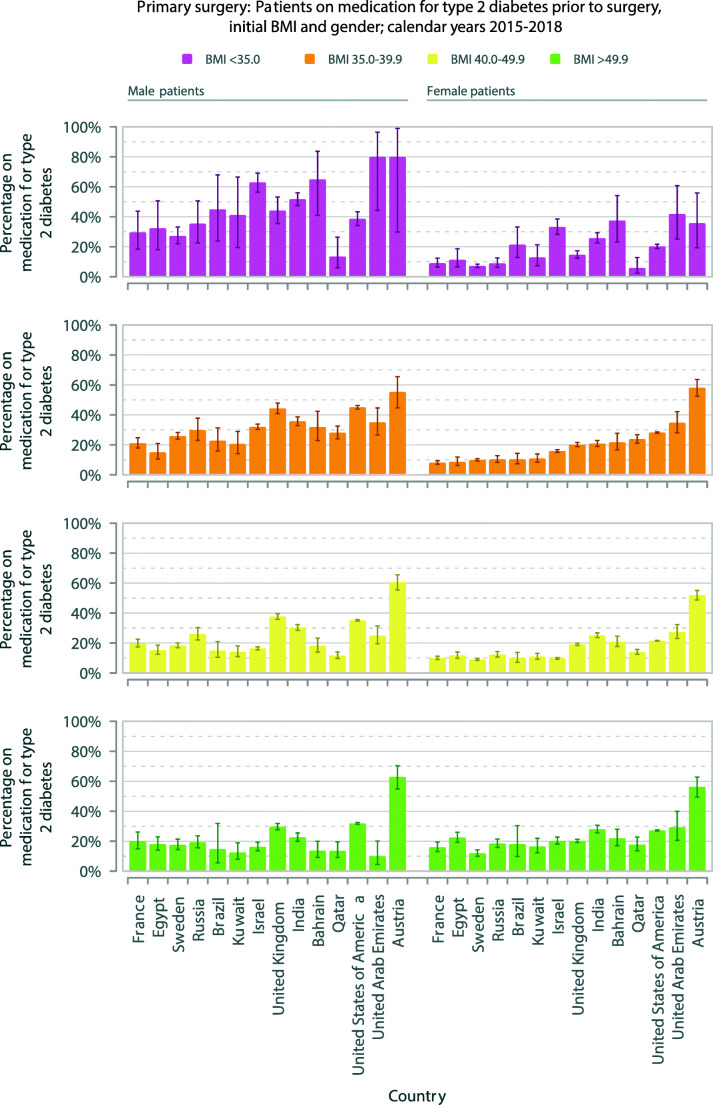
Primary surgery: male and female patients on medication for T2DM prior to surgery and initial BMI; calendar years 2015–2018. Data are rates (error bars 95% CI) ordered by increasing rate of T2DM per country for BMI range 35.0–39.9 kg/m^2^ in women. T2DM, type 2 diabetes mellitus; BMI, body mass index

The proportions of operated patients with T2DM varied widely from country to country. Sweden (12.0%) and France (12.3%) had the lowest, and Austria (55.1%) had the highest proportion of patients with T2DM (Fig. 1, Supplementary file Table 1).

The proportions of patients with T2DM in each obesity class are shown (Fig. 1, Supplementary file Table 1). A larger proportion of men were represented in the lower BMI classes, OR for BMI <35 kg/m^2^ compared to ≥35.0 kg/m^2^ 2.76 (2.52–3.03), *p* < 0.001 (Supplementary file Tables 2, 3). For women the equivalent OR was less than 1 with higher rates of T2DM in higher BMI ranges, OR 0.78 (0.73–0.83), *p* < 0.001. The USA contributed 72% of the patients, and when the remaining 28% of patients were analysed separately, the trends still remained for men, OR 2.47 (2.22–2.75), and for women, OR 0.85 (0.78–0.92), both *p* < 0.001.)

In 9 of 14 countries there were proportionately more men with T2DM compared to women with T2DM (*p* < 0.001). In Bahrain, Egypt, Kuwait Qatar, and UAE proportions were similar (Table 1). Overall, men had 68% greater odds for having T2DM compared to women (32%/21.9%, OR 1.68 (1.65–1.71), *p* < 0.001, Supplementary file Tables 2, 3). This pattern was not altered by excluding the USA data, OR 1.91 (1.85–1.97), *p* < 0.001).

### Proportions of Operated Patients with T2DM Compared to Country T2DM Prevalence

**Fig. 2:**
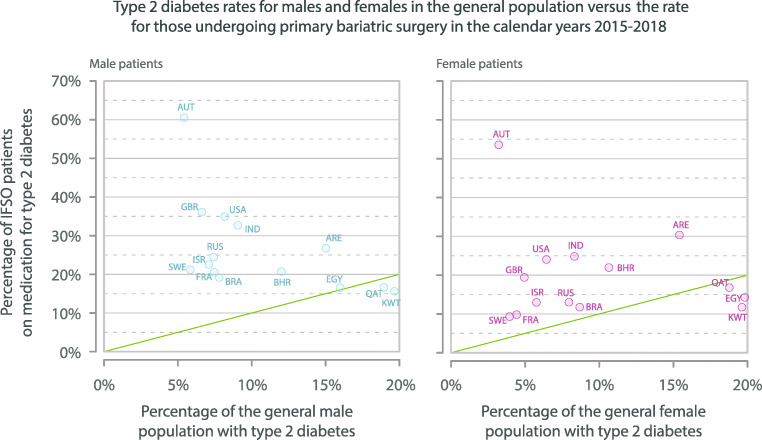
T2DM rates for adult men and women in the general adult (age ≥ 18 years) population from NCD-RisC, calendar year 2014, versus the rate for patients on medication for T2DM undergoing primary bariatric surgery, calendar years 2015–2018. Oblique line represents parity for general adult population prevalence of T2DM and proportion of operated patients on medication for T2DM. T2DM, type 2 diabetes mellitus; NCD-RisC, Non-Communicable Diseases Risk Factor Collaboration; AUT, Austria; BHR, Bahrain; BRA, Brazil; EGY, Egypt; FRA, France; IND, India; ISR, Israel; KWT, Kuwait; QAT, Qatar; RUS, Russia; SWE, Sweden; ARE, United Arab Emirates; GBR, United Kingdom; USA, United States of America

The proportion of operated male and female patients with T2DM for each country is shown compared to the individual country prevalence of T2DM (adults age ≥ 18 years) (Fig. 2, Supplementary file Table 4). Countries have been ordered by an increased prevalence of T2DM and a prevalence line indicated in Fig. 2. For Austria the proportion of patients with T2DM choosing surgery was well above the national prevalence rate for both sexes: 60.5% (56.6–64.4%) men had T2DM, 53.6% (51.1–56.1%) women had T2DM. In contrast for 2 countries, the proportions choosing surgery were below the prevalence rates for men, and for 3 countries, they were below the prevalence rates for women.

### Procedure Performed Based on T2DM Status

**Fig. 3:**
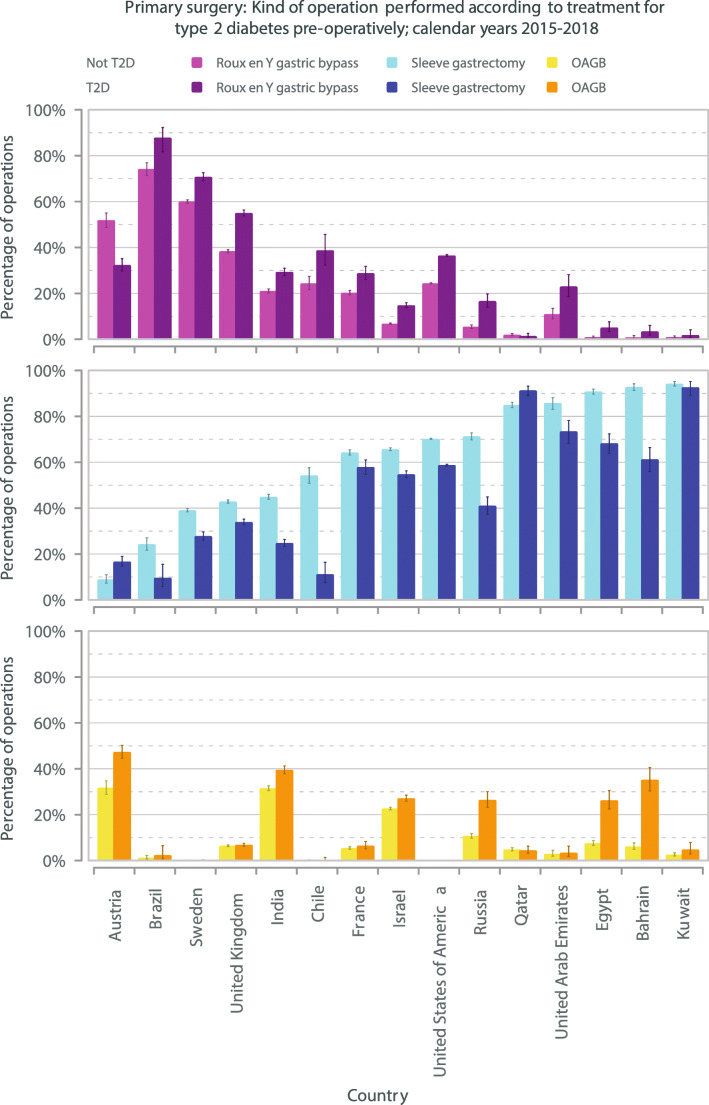
Primary surgery: type of operation performed for patients with or without medication for T2DM pre-operatively; calendar years 2015–2018, ordered by increasing rate of SG for patients without medication for T2DM. Data are rates (error bars 95% CI). T2DM, type 2 diabetes mellitus; SG, sleeve gastrectomy

Together, RYGB, OAGB and SG comprised 94.4% of total procedures for those without T2DM and 95.3% for those with T2DM (Supplementary file Table 5). For those without T2DM the individual operations comprised RYGB 25.1%, OAGB 3.4% and SG 65.8%. For those with T2DM the individual operations comprised RYGB 36.4%, OAGB 4.0% and SG 54.9%, OR 0.63 (0.63–0.64), *p* < 0.001 for SG vs non-SG for patients with T2DM. Twelve of 15 countries had higher proportions of gastric bypass (RYGB or OAGB) compared to SG operations for patients with T2DM compared to those without T2DM (Fig. 3/Supplementary file Table 5), OR 1.70 (1.67–1.72), *p* < 0.001. The equivalent OR with USA data excluded was 1.94 (1.88–2.00), *p* < 0.001. There were no significant differences for Austria, Kuwait or Qatar.

## Discussion

We describe the age, sex, BMI, relative proportions of operations per obesity class, and the operations undertaken for patients with T2DM compared to those without T2DM having primary bariatric-metabolic surgery in 15 countries in the IFSO Global Registry. Fewer men underwent surgery than women, and patients with T2DM were 7.4 years older than those without T2DM. Overall, regardless of the presence of T2DM, men were older than women and were proportionately more likely to have T2DM. In the majority of countries, particularly the 5 Middle Eastern countries, SG was the main operation for all patients irrespective of T2DM status, consistent with other reports [23]. However, T2DM was associated with a higher likelihood of diversionary surgery, either RYGB or OAGB, in nearly every country.

It has been shown previously that offering surgery to treat metabolic disease or diabetes rather than as a mere weight-reduction therapy changes demographic and clinical characteristics of surgical candidates [11]. Thus, men in our study appear more likely to seek surgery once they have developed T2DM. The presentation of patients with T2DM at an older age for surgery is in keeping with the increasing population prevalence for the disease with increasing age [27, 28]. Only 22.9% of the patients overall were men. On a population basis, the NCD-RisC data for BMI groups indicate that about one third of those with a BMI >35 kg/m^2^ are men in the countries studied [26]. This may provide some explanation for the relatively few men having bariatric-metabolic surgery. Many other large population-based studies have reported relative lack of uptake by men for bariatric-metabolic surgery [29–31]. A similar sex pattern emerges for recruiting participants into RCTs for T2DM comparing weight loss drugs. Sixty-four percent of those recruited for a liraglutide RCT as a therapy for T2DM were men [32], in contrast only 32% of those recruited for a liraglutide weight loss RCT were men [33].

The reasons for the differences in BMI for patients with or without T2DM in different countries are not known. Several international guidelines have lowered the BMI-based eligibility threshold down to 30 kg/m^2^ specifically for T2DM, and lower for Asians [9–11]. Especially for male patients, there was evidence that metabolic surgery for T2DM was being taken up in every country for class 1 obesity (Fig. 1). However, the smaller proportion of women with T2DM compared to men with T2DM suggests sex-specific motivational factors for choosing bariatric-metabolic surgery for class 1 obesity.

A previous study has shown extremely low uptake worldwide for bariatric-metabolic surgery for patients with obesity and T2DM [34]. For both sexes, in most countries the proportions receiving surgery were above the national prevalence line for T2DM prevalence, as shown in Fig. 2. However, the NCD-RisC T2DM prevalence data were for the general population, and not specifically for those with BMI > 35 kg/m^2^ and an average age of over 49 years, for which the prevalence would be much higher. Country-specific data for T2DM in the BMI-eligible population are lacking. However, the observation that the rates of surgery were below the national prevalence line for both sexes in some countries suggests a bias for those without diabetes to be offered and/or choose bariatric-metabolic surgery. Meanwhile, the countries with a level well above the line, for instance Austria, suggest a national trend to offer surgery for T2DM.

SG and RYGB are established procedures for T2DM, and IFSO supports OAGB as an effective procedure for this disease [35]. Although SG was the dominant operation for all patients, the higher usage of RYGB or OAGB for those with T2DM in 12 of 15 countries was significant. Removing from analysis the country contributing the majority of patients (USA) made the effect even more marked. The higher prevalence of bypass operations is in agreement with the literature. Among the 4 RCTs that compared SG and RYGB, only one had T2DM remission as an endpoint and was favourable to RYGB [2–5].

An overarching goal of the IFSO Global Registry is to achieve complete descriptive coverage of international bariatric practice, similar to existing reports of global obesity prevalence [36]. Therefore, future reports may be able to describe changing trends over time in the patients receiving bariatric-metabolic surgery, or differences in the operation done according to the severity or duration of disease in the whole operated population. Standardised international datasets recording anti-diabetes treatments would enable this. Other priorities for external validity of the international registry include verification of accuracy in submitted records and complete case ascertainment.

A strength of the study is the large number of patients and countries analysed, with individual-level actual data not based on estimates or surveys. Another strength is the more than 99% data completion rate for baseline T2DM albeit based on taking medications for diabetes.

Our study has several limitations. We are unable to assess whether the data are representative of individual country practice, except for Sweden, USA and Israel that have known near complete case ascertainment [30, 31, 37, 38]. For the other national registries data submission was not compulsory and it is unknown what proportion of practices submitted data. We are unable to exclude unknown observer or selection bias. Another limitation is that the reason(s) for patients choosing to have bariatric-metabolic surgery were not included in the dataset. Therefore it is not known whether patients with T2DM chose to have surgery for weight loss or to improve metabolic status. We also do not know whether operation choices were influenced by severity of T2DM. Due to the lack of individual country data on the prevalence of T2DM in individuals with BMI > 35 kg/m^2^, we were also unable to assess prioritisation of patients for metabolic surgery more accurately. Stratification by public health service or insurance/private funding was beyond the scope of the present study. As our data were cross-sectional we were unable to estimate changes over time. Another limitation is that the duration of T2DM was not recorded in the dataset.

## Conclusions

We found major differences in age, sex and uptake in patients with T2DM having bariatric-metabolic surgery compared to the operated population without T2DM, associated with a significant increased likelihood of a form of gastric bypass for patients with T2DM. Surgery for T2DM, or metabolic surgery, changes the landscape of the patient demographics. Overall, SG still predominated. The data could provide a knowledge base for healthcare systems considering frameworks for treating patients with T2DM.

## Supplementary Information

ESM 1 (DOCX 104 kb)

ESM 2 (DOCX 109 kb)

ESM 3 (DOCX 106 kb)

ESM 4 (DOCX 94 kb)

ESM 5 (DOCX 103 kb)

## Declarations

This article does not contain any studies with human participants or animals performed by any of the authors. For this type of study, formal consent was not required. A certificate of exemption from NHS Research Ethics Committee (REC) approval for the study was obtained from the UK Human Research Authority decision tools available on the website http://www.hra-decisiontools.org.uk/ethics/.
