# Supplementary material for: Bariatric-Metabolic Surgery Utilisation in Patients With and Without Diabetes: Data from the IFSO Global Registry 2015–2018
Source: Obes Surg. 2021 Feb 27;31(6):2391–400. doi: 10.1007/s11695-021-05280-6 (PMC8113173; doi:10.1007/s11695-021-05280-6)
Supplement: Supplementary file 1 — (DOCX 104 kb) [file 11695_2021_5280_MOESM1_ESM.docx]

**Supplementary material to Bariatric/Metabolic surgery uptake, selection of procedures and baseline diabetes data of a global registry**

**Table 1. Primary surgery for male and female patients 2015-2018: Rates of patients on medication for T2DM per BMI group ^a^**

|  | **Rate of patients on medication for T2DM** | | | | |
| --- | --- | --- | --- | --- | --- |
| **Country** | **BMI <35·0 kg/m^2^** | **BMI 35·0-39·9 kg/m^2^** | **BMI 40·0-49·9 kg/m^2^** | **BMI >49·9 kg/m^2^** | **P value *** |
| **Austria** | 42·4% (14/33) | 56·9% (234/411) | 54·0% (742/1373) | 57·9% (227/392) | 0·13 |
| Bahrain | 46·7% (28/60) | 24·7% (80/324) | 20·0% (166/831) | 18·4% (75/407) | <0·001 |
| **Brazil** | 26·7% (24/90) | 13·8% (62/450) | 11·8% (63/533) | 16·8% (6/95) | 0·002 |
| Chile | 24·3% (86/354) | 17·2% (88/513) | 20·0% (39/195) | 11·1% (1/9) | 0·068 |
| **Egypt** | 15·9% (25/157) | 10·7% (68/635) | 13·0% (196/1505) | 21·0% (196/934) | <0·001 |
| **France** | 11·5% (51/442) | 10·9% (297/2723) | 12·4% (496/3991) | 17·1% (128/750) | <0·001 |
| **India** | 37·5% (454/1211) | 26·9% (720/2680) | 27·4% (1421/5194) | 25·7% (544/2117) | <0·001 |
| **Israel** | 45·3% (262/578) | 20·8% (1887/9086) | 11·9% (1873/15740) | 18·8% (349/1858) | <0·001 |
| **Kuwait** | 16·9% (20/118) | 12·7% (84/659) | 11·9% (163/1372) | 15·0% (62/412) | 0·20 |
| Qatar | 8·4% (13/155) | 25·3% (332/1312) | 13·3% (372/2793) | 16·1% (76/472) | <0·001 |
| **Russia** | 12·2% (48/395) | 13·8% (124/900) | 15·9% (280/1757) | 18·7% (220/1175) | 0·003 |
| **Sweden** | 9·4% (219/2322) | 13·1% (987/7525) | 11·4% (1079/9428) | 13·8% (198/1430) | 0·28 |
| United Arab Emirates | 51·2% (21/41) | 35·1% (105/299) | 26·5% (156/589) | 21·0% (34/162) | <0·001 |
| **United Kingdom** | 18·7% (175/934) | 25·1% (969/3863) | 22·9% (2972/13003) | 22·5% (1704/7563) | <0·001 |
| **United States of America** | 22·5% (804/3578) | 31·1% (12300/39583) | 24·1% (35355/146565) | 28·4% (24533/86471) | <0·001 |
| All | 21·4% (n=2,244/10,468) | 25·8% (n=18,337,70,963) | 22·1% (n=45,373/204,869) | 27·2% (n=28,363/104,247) | <0·001 |

^a^ Bold indicates national registry. T2DM type 2 diabetes mellitus, BMI body mass index. * P value denotes multiple group chi^2^ test between the 4 BMI groups. Note denominators vary due to data completion rate.
