# Supplementary material for: Bariatric-Metabolic Surgery Utilisation in Patients With and Without Diabetes: Data from the IFSO Global Registry 2015–2018
Source: Obes Surg. 2021 Feb 27;31(6):2391–400. doi: 10.1007/s11695-021-05280-6 (PMC8113173; doi:10.1007/s11695-021-05280-6)
Supplement: Supplementary file 2 — (DOCX 109 kb) [file 11695_2021_5280_MOESM2_ESM.docx]

**Table 2. Primary surgery for male patients 2015-2018: Rates of patients on medication for T2DM per BMI group ^a^**

|  | **Rate of patients on medication for T2DM: males** | | | | | |
| --- | --- | --- | --- | --- | --- | --- |
| **Country** | **All operations** | **BMI <35·0 kg/m^2^** | **BMI 35·0-39·9 kg/m^2^** | **BMI 40·0-49·9 kg/m^2^** | **BMI >49·9 kg/m^2^** | **P value *** |
| **Austria** | 60·5% (382/631) | 80·0% (4/5) | 55·3% (52/94) | 60·6% (226/373) | 62·9% (100/159) | 0·53 |
| Bahrain | 20·7% (118/570) | 65·0% (13/20) | 31·9% (30/94) | 18·1% (51/281) | 13·7% (24/175) | <0·001 |
| **Brazil** | 19·2% (71/370) | 45·0% (9/20) | 22·8% (28/123) | 15·0% (29/193) | 14·7% (5/34) | 0·007 |
| **Egypt** | 16·5% (181/1,094) | 32·4% (11/34) | 15·1% (30/199) | 15·2% (84/551) | 18·1% (56/310) | 0·054 |
| **France** | 20·5% (375/1,826) | 29·6% (16/54) | 21·1% (122/577) | 19·8% (192/968) | 19·9% (42/211) | 0·36 |
| **India** | 32·7% (1,599/4,894) | 51·7% (283/547) | 35·7% (386/1,081) | 30·3% (669/2,207) | 22·6% (202/895) | <0·001 |
| **Israel** | 22·6% (1,967/8,721) | 63·0% (148/235) | 32·1% (875/2,728) | 16·4% (833/5,075) | 16·3% (111/683) | <0·001 |
| **Kuwait** | 15·6% (108/692) | 41·2% (7/17) | 20·6% (26/126) | 14·1% (55/389) | 12·5% (20/160) | 0·005 |
| Qatar | 16·6% (265/1,593) | 13·5% (7/52) | 28·1% (127/452) | 11·7% (106/905) | 13·6% (25/184) | <0·001 |
| **Russia** | 24·5% (260/1,062) | 35·4% (17/48) | 29·9% (47/157) | 26·0% (120/462) | 19·2% (76/395) | 0·007 |
| **Sweden** | 21·2% (983/4,636) | 27·2% (70/257) | 26·0% (381/1,468) | 18·4% (447/2,428) | 17·6% (85/483) | <0·001 |
| United Arab Emirates | 26·8% (110/411) | 80·0% (8/10) | 35·1% (40/114) | 25·0% (54/216) | 10·0% (7/70) | <0·001 |
| **United Kingdom** | 36·1% (1,946/5,389) | 44·2% (57/129) | 44·3% (348/785) | 37·7% (991/2,631) | 29·7% (546/1,838) | <0·001 |
| **United States of America** | 35·0% (21,664/61,979) | 38·6% (170/440) | 45·1% (3,006/6,667) | 35·1% (9,971/28,383) | 31·9% (7,074/22,208) | <0·001 |
| All | 32·0% (30,029/93,868) | 43·9% (820/1,868) | 37·5% (5,498/14,665) | 30·7% (13,828/45,062) | 30·1% (8,373/27,805) | <0·001 |

^a^ Raw data for Figure 1. Bold indicates national registry. T2DM type 2 diabetes mellitus, BMI body mass index. * P value denotes males multiple group chi^2^ test between the 4 BMI groups. Note denominators vary due to data completion rate.
