# Supplementary material for: Bariatric-Metabolic Surgery Utilisation in Patients With and Without Diabetes: Data from the IFSO Global Registry 2015–2018
Source: Obes Surg. 2021 Feb 27;31(6):2391–400. doi: 10.1007/s11695-021-05280-6 (PMC8113173; doi:10.1007/s11695-021-05280-6)
Supplement: Supplementary file 3 — (DOCX 106 kb) [file 11695_2021_5280_MOESM3_ESM.docx]

**Table 3. Primary surgery for female patients 2015-2018: Rates of patients on medication for T2DM per BMI group ^a^**

|  | **Rate of patients on medication for T2DM: females** | | | | | |
| --- | --- | --- | --- | --- | --- | --- |
| **Country** | **All operations** | **BMI <35·0 kg/m^2^** | **BMI 35·0-39·9 kg/m^2^** | **BMI 40·0-49·9 kg/m^2^** | **BMI >49·9 kg/m^2^** | **P value *** |
| **Austria** | 53·6% (832/1,553) | 35·7% (10/28) | 58·1% (182/313) | 51·9% (512/986) | 56·3% (126/224) | 0·047 |
| Bahrain | 22·0% (231/1,052) | 37·5% (15/40) | 21·7% (50/230) | 20·9% (115/550) | 22·0% (51/232) | 0·11 |
| **Brazil** | 11·8% (94/800) | 21·4% (15/70) | 10·4% (34/327) | 10·0% (34/340) | 18·0% (11/61) | 0·017 |
| **Egypt** | 14·3% (305/2,139) | 11·4% (14/123) | 8·7% (38/436) | 11·7% (112/954) | 22·4% (140/624) | <0·001 |
| **France** | 9·8% (604/6,153) | 9·0% (35/388) | 8·2% (175/2,146) | 10·1% (304/3,023) | 16·0% (86/539) | <0·001 |
| **India** | 24·8% (1,657/6,675) | 25·8% (171/664) | 20·9% (334/1,599) | 25·2% (752/2,987) | 28·0% (342/1,222) | <0·001 |
| **Israel** | 13·0% (2,404/18,541) | 33·2% (114/343) | 15·9% (1,012/6,358) | 9·8% (1,040/10,665) | 20·3% (238/1,175) | <0·001 |
| **Kuwait** | 11·8% (219/1,863) | 12·9% (13/101) | 10·9% (58/532) | 10·9% (107/980) | 16·5% (41/248) | 0·086 |
| Qatar | 16·8% (527/3,139) | 5·8% (6/103) | 23·8% (205/860) | 14·1% (265/1,886) | 17·7% (51/288) | <0·001 |
| **Russia** | 13·0% (412/3,165) | 8·9% (31/347) | 10·4% (77/743) | 12·4% (160/1,295) | 18·5% (144/780) | <0·001 |
| **Sweden** | 9·3% (1,500/16,069) | 7·2% (149/2,065) | 10·0% (606/6,057) | 9·0% (632/7,000) | 11·9% (113/947) | <0·001 |
| United Arab Emirates | 30·3% (206/679) | 41·9% (13/31) | 34·8% (64/184) | 27·4% (102/372) | 29·3% (27/92) | 0·16 |
| **United Kingdom** | 19·4% (3,879/19,992) | 14·7% (118/805) | 20·2% (621/3,078) | 19·1% (1,981/10,372) | 20·2% (1,158/5,725) | 0·001 |
| **United States of America** | 24·0% (56,415/234,783) | 20·2% (634/3,138) | 28·2% (9,294/32,916) | 21·5% (25,384/118,182) | 27·2% (17,459/64,263) | <0·001 |
| All | 21·9% (69,285/316,603) | 16·2% (1,338/8,246) | 22·9% (12,750/55,779) | 19·7% (31,500/159,592) | 26·2% (19,987/76,420) | <0·001 |

^a^ Raw data for Figure 1. Bold indicates national registry. T2DM type 2 diabetes mellitus, BMI body mass index. * P value denotes females multiple group chi^2^ test between the 4 BMI groups. Note denominators vary due to data completion rate.
