# Supplementary material for: Bariatric-Metabolic Surgery Utilisation in Patients With and Without Diabetes: Data from the IFSO Global Registry 2015–2018
Source: Obes Surg. 2021 Feb 27;31(6):2391–400. doi: 10.1007/s11695-021-05280-6 (PMC8113173; doi:10.1007/s11695-021-05280-6)
Supplement: Supplementary file 4 — (DOCX 94 kb) [file 11695_2021_5280_MOESM4_ESM.docx]

**Table 4. Country prevalence of T2DM and the rate for patients on medication for T2DM for those undergoing primary bariatric surgery, calendar years 2015-2018 ^a^**

|  | **Male** | | **Female** | |
| --- | --- | --- | --- | --- |
|  | **NCD-RisC Age-standardised diabetes prevalence (95% CI)** | **IFSO Global Registry (95% CI)** | **NCD-RisC Age-standardised diabetes prevalence (95% CI)** | **IFSO Global Registry (95% CI)** |
| Country | Rate | Rate | Rate | Rate |
| **Austria** | 5·4% (2·8-9·1) | 60·5% (56·6-64·4%) | 3·2% (1·6-5·7) | 53·6% (51·1-56·1%) |
| Bahrain | 12·0% (7-18·5) | 20·7% (17·5-24·3%) | 10·6% (6·1-16·7) | 22·0% (19·5-24·6%) |
| **Brazil** | 7·8% (4·1-13·3) | 19·2% (15·4-23·7%) | 8·7% (4·6-14·4) | 11·8% (9·6-14·2%) |
| **Egypt** | 16·0% (10-23·6) | 16·5% (14·4-18·9%) | 19·8% (12·9-28·2) | 14·3% (12·8-15·8%) |
| **France** | 7·5% (4·1-12·2) | 20·5% (18·7-22·5%) | 4·4% (2·3-7·5) | 9·8% (9·1-10·6%) |
| **India** | 9·1% (5·2-14·2) | 32·7% (31·4-34·0%) | 8·3% (4·8-12·8) | 24·8% (23·8-25·9%) |
| **Israel** | 7·1% (4-11·6) | 22·6% (21·7-23·4%) | 5·8% (2·8-10) | 13·0% (12·5-13·5%) |
| **Kuwait** | 19·7% (12·8-28·1) | 15·6% (13·0-18·6%) | 19·6% (12·9-27·7) | 11·8% (10·3-13·3%) |
| Qatar | 18·9% (12-27) | 16·6% (14·9-18·6%) | 18·8% (12·2-26·8) | 16·8% (15·5-18·2%) |
| **Russia** | 7·4% (3·4-13·1) | 24·5% (21·9-27·2%) | 8·0% (3·7-13·8) | 13·0% (11·9-14·3%) |
| **Sweden** | 5·8% (3·2-9·3) | 21·2% (20·0-22·4%) | 4·0% (2·1-6·6) | 9·3% (8·9-9·8%) |
| United Arab Emirates | 15·0% (9·2-22·5) | 26·8% (22·6-31·4%) | 15·4% (9·7-22·6) | 30·3% (26·9-34·0%) |
| **United Kingdom** | 6·6% (4·1-9·7%) | 36·1% (34·8-37·4%) | 4·9% (3·1-7·4) | 19·4% (18·9-20·0%) |
| **United States of America** | 8·2% (4·9-12·7%) | 35·0% (34·6-35·3%) | 6·4% (3·8-9·9) | 24·0% (23·9-24·2%) |

^a^ Raw data for Figure 2. Bold indicates national registry. T2DM type 2 diabetes mellitus, NCD-RisC Non-Communicable Disease Risk Factor Collaboration, 2014 data.
