# Supplementary material for: Bariatric-Metabolic Surgery Utilisation in Patients With and Without Diabetes: Data from the IFSO Global Registry 2015–2018
Source: Obes Surg. 2021 Feb 27;31(6):2391–400. doi: 10.1007/s11695-021-05280-6 (PMC8113173; doi:10.1007/s11695-021-05280-6)
Supplement: Supplementary file 5 — (DOCX 103 kb) [file 11695_2021_5280_MOESM5_ESM.docx]

**Table 5. Primary surgery: type of operation performed for patients with or without medication for T2DM pre-operatively; calendar years 2015-2018 ^a^**

|  | **No medication for T2DM** | | | | **Medication for T2DM** | | | |  |  |
| --- | --- | --- | --- | --- | --- | --- | --- | --- | --- | --- |
| **Country** | **RYGB** | **OAGB** | **SG** | **Other  / Unknown** | **RYGB** | **OAGB** | **SG** | **Other  / Unknown** | **P value** |  |
| **Austria** | 515 | 315 | 89 | 73 | 395 | 578 | 204 | 42 | 0·024 |  |
| Bahrain | 11 | 79 | 1182 | 1 | 12 | 123 | 214 | 0 | <0·001 |  |
| **Brazil** | 746 | 13 | 244 | 2 | 145 | 4 | 16 | 0 | <0·001 |  |
| Chile | 209 | 0 | 465 | 183 | 83 | 0 | 24 | 107 | <0·001 |  |
| **Egypt** | 25 | 209 | 2496 | 17 | 25 | 128 | 332 | 1 | <0·001 |  |
| **France** | 1422 | 383 | 4499 | 696 | 282 | 64 | 567 | 66 | <0·001 |  |
| **India** | 1751 | 2621 | 3736 | 205 | 956 | 1288 | 808 | 204 | <0·001 |  |
| **Israel** | 1542 | 5193 | 15034 | 1122 | 648 | 1187 | 2394 | 142 | <0·001 |  |
| **Kuwait** | 21 | 59 | 2106 | 48 | 6 | 16 | 305 | 2 | 0·011 |  |
| Qatar | 75 | 187 | 3259 | 420 | 11 | 35 | 706 | 41 | 0·42 |  |
| **Russia** | 194 | 383 | 2535 | 443 | 112 | 178 | 276 | 106 | <0·001 |  |
| **Sweden** | 10942 | 8 | 7123 | 149 | 1758 | 2 | 691 | 32 | <0·001 |  |
| United Arab Emirates | 85 | 23 | 665 | 2 | 73 | 11 | 233 | 0 | <0·001 |  |
| **United Kingdom** | 7501 | 1268 | 8389 | 2398 | 3206 | 402 | 1980 | 237 | <0·001 |  |
| **United States of America** | 53364 | 0 | 153530 | 11789 | 28515 | 0 | 45903 | 3661 | <0·001 |  |
| All | 78403 | 10741 | 205352 | 17548 | 36227 | 4016 | 54653 | 4641 | <0·001 | |

^a^ Raw data for Figure 3. Bold indicates national registry. T2DM type 2 diabetes mellitus, RYGB Roux en Y Gastric Bypass, OAGB one anastomosis gastric bypass, SG sleeve gastrectomy. * P value denotes RYGB and OAGB combined compared to non-bypass for no medication for T2DM vs medication for T2DM, chi^2^ test.
